# PD-1 and LAG-3: synergistic fostering of T cell exhaustion

**DOI:** 10.1038/s41392-024-02000-1

**Published:** 2024-10-18

**Authors:** Maike Hofmann, Robert Thimme, Wolfgang W. Schamel

**Affiliations:** 1 Department of Medicine II, Medical Center, Faculty of Medicine, University of Freiburg, Freiburg, Germany; 2 Signaling Research Centers BIOSS and CIBSS, University of Freiburg, Freiburg, Germany; 3 Department of Immunology, Faculty of Biology, University of Freiburg, Freiburg, Germany; 4 Centre for Chronic Immunodeficiency (CCI), Faculty of Medicine, University of Freiburg, Freiburg, Germany

**Keywords:** Immunotherapy, Cell biology

In the August issue of *Cell* 2024, three back-to-back papers disentangled non-redundant inhibitory mechanisms of PD-1 and LAG-3 in exhausted murine and human CD8+ T cells.^1–3^ These findings will have a clear impact on checkpoint blockade therapies against a variety of tumors.

**Fig. 1:**
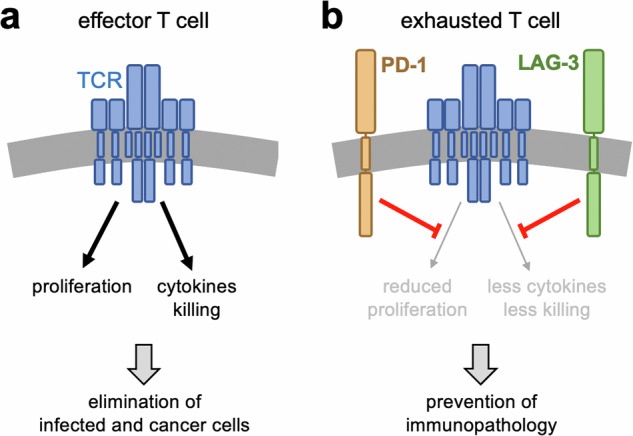
PD-1 and LAG-3 play different roles in inhibiting T cell activation in exhausted T cells. **a** In effector CD8+ T cells, stimulation of the TCR and co-receptors (not shown) leads to the proliferation of the cells and the execution of effector programs, such as the secretion of cytokines and the killing of infected cells or tumor cells. This leads to the control of the infection and cancer. **b** Exhausted CD8+ T cells are characterized by the expression of inhibitory receptors, such as PD-1 and LAG-3. Signaling by PD-1 specifically reduces TCR- and co-receptor-induced proliferation. Complementary, signals by LAG-3 diminish the production of cytokines and the killing of target cells. Thus, the CD8+ T cell does neither proliferate nor get activated, therefore preventing to harm healthy tissues

CD8+ T cells, also called cytotoxic T cells, are major effector cells of the immune response, killing infected and malignant cells. Upon activation through the T cell receptor (TCR) and co-receptors, these T cells produce cytokines and cytotoxic molecules to promote an inflammatory environment and induce cell death (Fig. 1a). An excessive CD8+ T cell response can lead to significant damage to the host by harming healthy tissue in processes called immunopathology and autoimmunity. This is especially relevant in persisting stimulation of CD8+ T cells during chronic viral infection and cancer. Thus, CD8+ T cell activation must be balanced to ensure control of pathogens and cancer while limiting overwhelming pathology. Therefore, numerous regulatory mechanisms exist to keep the CD8+ T cell response in balance.^4^

T cell exhaustion represents such a regulatory mechanism of CD8+ T cells undergoing chronic stimulation. Exhausted CD8+ T cells are characterized by expression of the key transcription factor TOX and inhibitory receptors, as well as limited effector functions, reduced proliferation, altered metabolism, distinct transcriptomic and epigenetic profiles.^5,6^ The molecular features of exhausted T cells have been intensively investigated in recent years, such as prolonged expression of multiple inhibitory receptors, including PD-1 and LAG-3. However, how these different receptors inhibit the activating signals by the TCR and co-receptors and thereby regulate T cell exhaustion is not well understood.

Ngiow et al.^1^ addressed the interaction of PD-1 and LAG-3 in the mouse model of chronic lymphocytic choriomeningitis virus (LCMV) infection by a quadruple adoptive transfer strategy. They injected wildtype, PD-1 knock out (KO), LAG-3 KO and PD-1/LAG-3 double KO CD8+ T cells at a ratio of 1:1:1:1 into the same recipient mouse. These T cells had a transgenic TCR to recognize a peptide from LCMV. Chronic infection of these mice with LCMV represents the prototype model for studying T cell exhaustion and key underlying mechanisms have been identified using this model system. Now, using chronic LCMV infection, the authors carefully analyzed the four types of T cells. They confirmed the PD-1-mediated restriction of exhausted CD8+ T cell proliferation. In addition, they uncovered that LAG-3 specifically represses the effector function of exhausted CD8+ T cells, such as cytokine production and cytotoxicity (Fig. 1b). By this, a synergistic role of PD-1 and LAG-3 in the development of exhausted CD8+ T cells has been identified. Unexpectedly the data imply that the signals by the activating receptors leading to proliferation and effector function are distinct, since they can be separately inhibited by PD-1 and LAG-3, respectively. Mechanistically, LAG-3 is required to sustain expression of the exhaustion-promoting transcriptional regulator TOX and therefore orchestrates expression of inhibitory receptors and drives differentiation as well as maintenance of exhausted CD8+ T cells.

Moreover, the authors observed that the LAG-3/TOX pathway also regulates the expression of natural killer (NK) receptors on the exhausted CD8+ T cells. Indeed, LAG-3 expression is linked to increased expression of the inhibitory CD94/NKG2A receptor and repressed expression of the activating CD94/NKG2C receptor. Thus, LAG-3 controls a functional switch of the CD94/NK receptor pathways. Both, CD94/NKG2A and CD94/NKG2C bind the same ligands, namely, the non-classical MHC class I molecules, Qa-1 in mice and HLA-E in humans that are upregulated on stressed and malignant cells. Due to their binding to the inhibitory CD94/NKG2A receptor, Qa-1 and HLA-E are associated with tumor immune evasion. Thus, Ngiow et al.^1^ discovered a LAG-3/TOX/CD94 immunosurveillance axis that regulates the effector activity of exhausted CD8+ T cells toward stressed and tumor cells in the context of chronic stimulation.

Ngiow et al. also showed that CD94/NKG2 expression on exhausted CD8+ T cells can be recalibrated by injection of anti-LAG-3 and anti-PD-1 antibodies (called relatlimab and nivolumab, respectively) in mice and humans in the context of melanoma, proofing a therapeutic potential. Accordingly, the companion reports by Andrews et al. and Cillo et al.^2,3^ extent on this finding of recalibrated tumor immunosurveillance by CD8+ T cells after combined anti-PD-1/anti-LAG-3 treatment by demonstrating increased response to IFNγ in addition to TCR signaling. Enhanced tumor clearance and survival were evident in a melanoma mouse model and in metastatic melanoma patients following the treatment.^2,3^

Hence, relieving the blocks of proliferation and effector function (Fig. 1b), explain the beneficial effect of simultaneous targeting of both PD-1 and LAG-3 by combined nivolumab and relatlimab checkpoint blockade therapy in melanoma. Indeed, the clinical benefit has been clearly demonstrated in a phase 2–3, double-blind, randomized trial that evaluated relatlimab and nivolumab as a fixed-dose combination as compared with nivolumab alone in patients with previously untreated metastatic or unresectable melanoma.^7^ Inhibition of both immune checkpoints, LAG-3 and PD-1, provided a greater benefit with regard to progression-free survival (10.1 months (95% confidence interval [CI], 6.4–15.7)) with relatlimab–nivolumab as compared with 4.6 months (95% CI, 3.4–5.6) with nivolumab alone without showing new safety signals.^7^

Yet, open questions remain concerning the different signaling molecules or pathways that PD-1 and LAG-3 inhibit. In addition, several ligands for LAG-3 exist with different binding sites and cross-linking potential. It will be relevant to study whether different ligands modulate the signaling pathways differentially. In addition, it will also be instrumental to understand how the anti-PD-1 and anti-LAG-3 antibodies block PD-1/LAG-3 signaling with the implications for improving therapeutic strategies. Clearly, these three companion articles set the stage for increasing the effort to investigate the interconnection of instructing, driving and coordinating signals in CD8+ T cell exhaustion. By such future studies we will learn how to specify our combined checkpoint blockade approaches in malignancies affecting different organs in terms of efficacy and safety.
